# EEG Biomarkers Related With the Functional State of Stroke Patients

**DOI:** 10.3389/fnins.2020.00582

**Published:** 2020-07-07

**Authors:** Marc Sebastián-Romagosa, Esther Udina, Rupert Ortner, Josep Dinarès-Ferran, Woosang Cho, Nensi Murovec, Clara Matencio-Peralba, Sebastian Sieghartsleitner, Brendan Z. Allison, Christoph Guger

**Affiliations:** ^1^Department of Physiology, Universitat Autònoma de Barcelona, Barcelona, Spain; ^2^g.tec Medical Engineering Spain SL, Barcelona, Spain; ^3^Data and Signal Processing Research Group, Department of Engineering, University of Vic - Central University of Catalonia, Vic, Spain; ^4^g.tec Medical Engineering GmbH, Schiedlberg, Austria; ^5^Department of Cognitive Science, University of California at San Diego, La Jolla, CA, United States

**Keywords:** brain-computer interface, motor imagery, EEG, rehabilitation, Brain Symmetry Index, laterality coefficient

## Abstract

**Introduction:**

Recent studies explored promising new quantitative methods to analyze electroencephalography (EEG) signals. This paper analyzes the correlation of two EEG parameters, Brain Symmetry Index (BSI) and Laterality Coefficient (LC), with established functional scales for the stroke assessment.

**Methods:**

Thirty-two healthy subjects and thirty-six stroke patients with upper extremity hemiparesis were recruited for this study. The stroke patients where subdivided in three groups according to the stroke location: Cortical, Subcortical, and Cortical + Subcortical. The participants performed assessment visits to record the EEG in the resting state and perform functional tests using rehabilitation scales. Then, stroke patients performed 25 sessions using a motor-imagery based Brain Computer Interface system (BCI). BSI was calculated with the EEG data in resting state and LC was calculated with the Event-Related Synchronization maps.

**Results:**

The results of this study demonstrated significant differences in the BSI between the healthy group and Subcortical group (*P* = 0.001), and also between the healthy and Cortical+Subcortical group (*P* = 0.019). No significant differences were found between the healthy group and the Cortical group (*P* = 0.505). Furthermore, the BSI analysis in the healthy group based on gender showed statistical differences (*P* = 0.027). In the stroke group, the correlation between the BSI and the functional state of the upper extremity assessed by Fugl-Meyer Assessment (FMA) was also significant, ρ = −0.430 and *P* = 0.046. The correlation between the BSI and the FMA-Lower extremity was not significant (ρ = −0.063, *P* = 0.852). Similarly, the LC calculated in the alpha band has significative correlation with FMA of upper extremity (ρ = −0.623 and *P* < 0.001) and FMA of lower extremity (ρ = −0.509 and *P* = 0.026). Other important significant correlations between LC and functional scales were observed. In addition, the patients showed an improvement in the FMA-upper extremity after the BCI therapy (ΔFMA = 1 median [IQR: 0–8], *P* = 0.002).

**Conclusion:**

The quantitative EEG tools used here may help support our understanding of stroke and how the brain changes during rehabilitation therapy. These tools can help identify changes in EEG biomarkers and parameters during therapy that might lead to improved therapy methods and functional prognoses.

## Introduction

Stroke is one of the most prevalent pathologies around the world. Stroke can cause devastating effects in survivors, including severe motor and sensory impairments that hinder their activities of daily living (Kim et al., 2020). The clinical consequences after a stroke vary, depending largely on the location and the cause of the damage (Prabhakaran et al., 2008). Diagnostic imaging tools like Computational Tomography (CT) or Functional magnetic resonance imaging (fMRI) are normally used to evaluate brain damage in the acute and sub-acute phases, offering valuable information about the diagnostic and functional prognosis for each case. Recent studies explored new methods to process and analyze brain signals acquired by conventional techniques like electroencephalography (EEG) (Kanda et al., 2009; Leon-Carrion et al., 2009; Foreman and Claassen, 2012; Rabiller et al., 2015; Wu et al., 2016) or magnetoencephalography (MEG) (Mäkelä et al., 2015; Ikkai et al., 2016; Krauss et al., 2018).

Quantitative EEG (qEEG) is a useful tool to extract features from the EEG signals and thereby help clinicians understand each patient’s clinical state. qEEG parameters have shown multiple correlations with different pathologies, making qEEG an essential tool for different clinical fields (Nishida et al., 2011; Wang et al., 2013; Faust et al., 2015; Jeong et al., 2016; Muniz et al., 2016; Piano et al., 2017). One qEEG parameter is the Brain Symmetry Index (BSI), described by van Putten et al. (2004) to assess the stroke risk during carotid endarterectomy surgery in real time (van Putten et al., 2004; van Putten, 2006, 2007). Subsequently, Agius Anastasi et al. (2017) used the BSI with stroke patients and found correlations between this parameter and functional scales. The idea of the BSI is to assess the symmetry between both brain hemispheres by using the EEG.

EEG can measure brain signals with a high temporal resolution, allowing clinicians to monitor brain activity in real time (Dobkin, 2007; McFarland and Wolpaw, 2017). Brain signals can be read with a software program to provide the user with an external pathway for these brain outputs (Wolpaw, 2007). This approach has been employed in numerous Brain Computer Interface (BCI) systems providing real-time communication and control. BCIs have been used to control devices such as a wheelchair (Carlson and del, 2013), prosthesis or functional electrical stimulator (FES) (Ramos-Murguialday et al., 2013), sometimes in combination with immersive feedback relating to rehabilitation (Shokur et al., 2018). Over the past several years, many publications have combined BCI, FES and other feedback devices to increase cortical plasticity in stroke survivors helping them regain movement control (Dobkin, 2007).

In this approach to movement restoration, stroke survivors perform Motor Imagery (MI) exercises during EEG recording (Cervera et al., 2018). The decoded brain oscillations can be used to move a virtual reality avatar or trigger an FES device to reproduce the imagined movement with the paretic limb (e.g., Cho et al., 2016; Irimia et al., 2017). These types of rewarding feedback only occur if the patient imagines the desired movement, providing a closed-loop feedback system for patients and an objective means to monitor patient compliance for therapists and scientists.

During the MI tasks, the patient should concentrate on imagining a specific movement instructed by a therapist, such as wrist dorsiflexion. During MI, the contralateral motor cortex will exhibit event-related desynchronization (ERD), which is a decrease of EEG bandpower in the μ (8–13 Hz) and β (16–30 Hz) range. After the patient finishes performing MI, the contralateral motor cortex exhibits an increase in μ and lower β rhythm activity, called event-related synchronization (ERS). An ERS can also occur during MI in the ipsilateral hemisphere in the μ range, and is related to an idling state of those areas (Pfurtscheller and Aranibar, 1979; Graimann et al., 2002; Neuper et al., 2006; Kaiser et al., 2012). Many people with stroke exhibit atypical ERD/ERS activation patterns; for example, the affected cortex may be less excitable, and the changes in EEG activity may be more prominent over nearby cortical areas (Neuper et al., 2006; Kaiser et al., 2012).

Hence, stroke patients often have abnormal changes in ERD/ERS patterns resulting from MI. Kaiser et al. (2012) investigated how these abnormal patterns relate to the patient’s functional state and spasticity, using a new parameter, the Laterality Coefficient (LC). For physical assessment, they used the European Stroke Scale (ESS), the Medical Research Council (MRC) and the Modified Ashworth Scale (MAS). The LC presented significant correlations with the MRC scale and MAS. The findings of Kaiser et al. (2012) showed that high percentage changes in ERD patterns in the contralesional hemisphere are related to a high degree of impairment. However, the aim of the current publication is to evaluate a novel analysis studying the correlation of LC with other functional scales like the Fugl-Meyer assessment.

Here, we explore two different qEEG parameters and their relationship with the diagnosis and functional prognosis of stroke patients. One group of healthy participants and one group of stroke patients participated in the study. Stroke patients performed functional assessment sessions, and BCI rehabilitation therapy for the upper extremity. EEG was recorded in two different situations: 8 min of resting state with open eyes (rEEG), and MI using a BCI system for motor rehabilitation. BSI perameters were analyzed with rEEG, whereas LC was calculated during the MI period. To assess each patient’s functionality before and after the therapy, we primarily used the Fugl-Meyer assessment (FMA) (Gladstone et al., 2002; Woytowicz et al., 2017). We also used eight other standardized tests used in rehabilitation to assess motor function, spasticity, cognitive function, and other parameters: Fahn Tremor Rating Scale, MAS, Barthel Index, Box and Block Test, 9 Hole Peg test, 2 Point Discrimination Test, Montreal Cognitive Assessment and Self-rated questionnaire. This is the first study to employ such a broad range of tests along with analyses of BSI and other EEG-based parameters across several therapy sessions.

## Materials and Methods

### Participants

Thirty-two healthy subjects and thirty-six stroke patients with upper extremity hemiparesis were recruited for this study. All healthy participants were volunteers recruited through the Universitat de Vic, Spain. The stroke patients were recruited in the rehabilitation center RecoveriX Gym in Schiedlberg, Austria. Two patients dropped out from the study because of personal problems that prevented them from attending recording sessions. The patients’ characteristics are reported in the results section.

The inclusion criteria for stroke patients were: (i) residual hemiparesis, (ii) the stroke occurred at least 4 days before the first assessment, (iii) functional restriction in the upper extremities. Additionally, for all participants, the following criteria were applied: (iv) able to understand written and spoken instructions, (v) stable neurological status, (vi) willing to participate in the study and to understand and sign the informed consent, (vii) able to attend recording sessions, (viii) no cerebellar lesions, (ix) no Botulinum toxin treatment for spasticity during the study. The participants were not recruited based on the scores of any functional assessment. Ethics approval was obtained from the Ethikkommission des Landes Oberösterreich in Austria for the patients (#D-42-17), and the ethics committee of Comitè d’Ètica de la Recerca-CER of Universitat de Vic (Spain) for the healthy controls.

### Protocol

#### Healthy Controls

The healthy controls sat in a comfortable chair for 8 min while rEEG was collected. During this resting state assessment, participants were asked to avoid unnecessary movements and keep their eyes open, aside from normal blinking.

#### Stroke Patients

Each stroke patient participated in four assessment sessions and 25 therapy sessions.

Assessment: A clinician assessed each patient twice before the therapy began and twice after the last therapy session. Each of these four assessment sessions had two components: (1) the clinician recorded 8 min of rEEG with the same settings as described above for healthy controls and (2) the clinician tested the patient’s motor function. The Pre1 assessment was performed 1 month before starting the therapy, and the Pre2 assessment was performed just before the therapy started. The Post1 assessment was performed just after the last session, and the Post2 assessment occurred 1 month after the last session. One hundred and and thirty six assessment sessions were performed in total (4 per patient).

Therapy: Patients completed 25 sessions, with two sessions per week.

**FIGURE 1:**
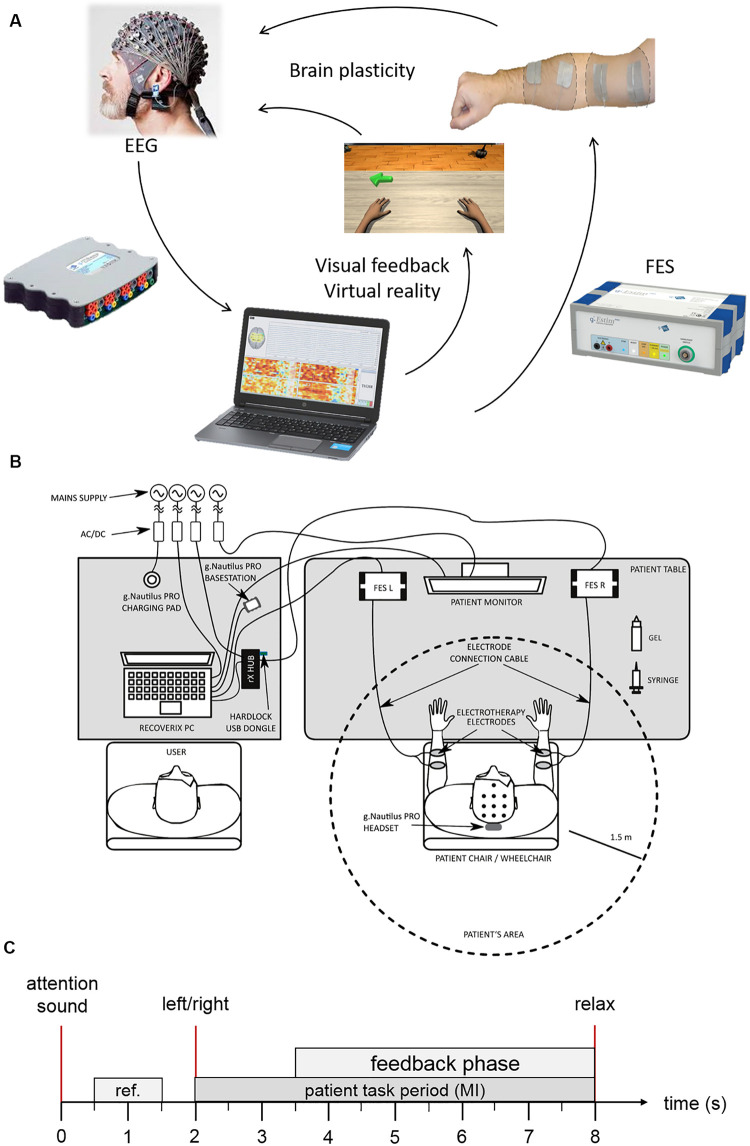
BCI system components. **(A)** Shows the motor learning loop. **(B)** System setup. **(C)** Trial description.

Figures 1A,B depict different system components and the physical layout during each therapy session. At the beginning of each therapy session, the therapist talked with each patient to confirm that the patient understands the MI task and the upcoming procedure. Next, the EEG cap and FES pads were placed on the patient, and FES parameters were adjusted, as detailed below. After this preparation, the patient was seated in a comfortable chair in front of a table, facing a monitor where two virtual arms were projected in a position and orientation mimicking the subject’s arms. The patient was asked to place both hands on the table and perform MI while following cues and feedback presented on the monitor. Each session contained up to three runs of 80 trials each, depending of the patient’s fatigue. At the end of each session, the cap and FES pads were removed, and the skin was cleaned with a moist cloth. Each session required about 60 min total, including preparation and cleaning.

### BCI System Description

The BCI system used in this study was RecoveriX (g.tec medical engineering GmbH, Austria) (Irimia et al., 2016). This system managed all EEG data recording and real-time interactions with the patient and therapist, including visual feedback using a virtual reality avatar and proprioceptive feedback using FES. Participants wore EEG caps with 16 active electrodes at positions FC5, FC1, FCZ, FC2, FC6, C5 C3, C1, CZ, C2, C4, C6, CP5, CP1, CP2, and CP6, according to the international 10/10 system (extended 10/20 system). A reference electrode was placed on the right earlobe and a ground electrode at FPZ.

Two FES electrodes were placed on the skin over the wrist extensors of the left and right forearms. The frequency was set to 50 Hz, and the pulse-width was set to 300 μs. Then, the stimulation parameters were individually adjusted for session until either (1) the optimal passive movement without pain for patients with mild or moderate muscle spasm, or (2) muscle contraction was observed in the target muscle of the paretic side for patients with severe muscle spasm.

#### MI Exercise

Figure 1C depicts the timing of each trial. Each trial starts with a beep, to help the participant focus on the upcoming task. Two seconds later, the system presents the instruction. For 1.5 s, the system presents an arrow pointing to the left or right on the patient’s monitor and the word “left” or “right” in the participant’s mother tongue via headphones. These simultaneous visual and auditory cues direct the patient to imagine dorsiflexion of the left or right wrist (in pseudorandom order). The participant is instructed to start the MI immediately after receiving the command and to continue the MI until the relax command is presented auditorily. The feedback phase starts at the same time as the instruction ends, that is 3.5 s after trial begin. The feedback devices can only be activated during this phase.

#### Feedback Presentation

Visual and proprioceptive FES feedback are provided in synchrony and only in the feedback phase. Classification of motor imagery is done every second. If the classified MI matches the presented command (left or right), then feedback is switched on, which means the wrist dorsiflexion is initiated by electrical stimulation and presented visually on the computer screen. During incorrect classifications, the initiated movement is done in the opposite direction. For classification, we used linear discriminant analysis (LDA) on the spatially filtered data. We followed the steps described in Irimia et al. (2018) except for a change in the electrode setup: FPZ, FP1, FP2, AF7, AF3, AFZ, AF4, AF8, F7, F5, F3, F1, FZ, F2, F4, F6, F8, FT7, FC5, FC3, FC1, FCZ, FC2, FC4, FC6, FT8, T7, C5, C3, C1, CZ, C2, C4, C6, T8, TP7, CP5, CP3, CP1, CPZ, CP2, CP4, CP6, TP8, P7, P5, P3, P1, PZ, P2, P4, P6, P8, PO7, PO3, POZ, PO4, PO8, O1, OZ, O2, O9, O10.

#### Event-Related Synchronization and Desynchronization

**FIGURE 2:**
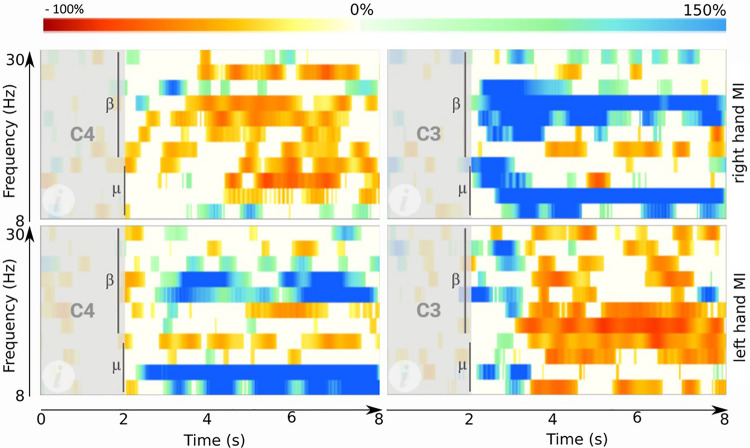
ERD maps obtained after one session of BCI training. **Top right:** ERD map during right hand MI on site C3. **Top left:** ERD map during right hand MI on C4. **Bottom right:** ERD map during left hand MI on C3. **Bottom left:** ERD map during left hand MI on C4. Each plot shows the time from 0 to 8 s (*x*-axis) and frequencies between 8 and 30 Hz (*y*-axis). Red areas indicate high ERD. Blue areas mark the opposite: an ERS. Vertical bars indicate the cue onset at 2 s.

Figure 2 presents ERD/ERS patterns that typically occur during MI. This figure was generated using the data from one BCI training session. During MI, the contralateral motor cortex produces a desynchronization (event-related desynchronization or ERD) of cortical motor neurons, showing a decrease in the bandpower of the waves with a frequency of 8–13 Hz (mu frequency rhythm). The ipsilateral motor cortex shows ERS patterns to suppress corresponding motor areas during MI of the opposite hand side (Pfurtscheller and Aranibar, 1979; Graimann et al., 2002; Neuper et al., 2006; Kaiser et al., 2012). To create such maps, the change of EEG bandpower of several bandpass filtered frequency bands was calculated and plotted. The frequency bands chosen here ranged from 8 to 30 Hz in steps of 2 Hz. In each band, the power was calculated stepwise in windows of 16 samples (0.0625 s). Then, the bandpower of each window was compared to the bandpower of the reference period (gray area in Figure 2), during which the participant was in a resting state. The comparison used the following formula, in which A is the bandpower of one single window and R the bandpower within the reference period:


$$E\,R\,D=\frac{A-R}{R}*100\%$$

Finally, a bootstrapping significance test (α = 0.05) was done for all windows. Values that are not significant were set to 0 and are plotted in white in Figure 2. High ERD values (decreased bandpower) are plotted in red, whereas high ERS values (increased bandpower) are plotted in blue.

### EEG Parameters

#### Brain Symmetry Index

The BSI is a parameter that compares the spectral power of the two hemispheres of the brain using bandpass filtered EEG signals. The BSI value ranges from 0 to 1 and is a measure of the symmetry between both hemispheres. A BSI value of 0 reflects total symmetry and 1 total asymmetry. The BSI value should be closer to 0 in healthy people and higher in stroke patients.

#### Method

The BSI of a segment of EEG is calculated using a revised BSI formula (van Putten, 2007), which is based on the squared value of the Fourier coefficients:


$$B\,S\,I\,\left(t\right)=\frac{1}{K}\,\sum_{n=1}^{K}\left|\frac{R_{n}^{*}\,\left(t\right)-L_{n}^{*}\,\left(t\right)}{R_{n}^{*}\,\left(t\right)+L_{n}^{*}\,\left(t\right)}\right|$$

with


$$R_{n}^{*}\,\left(t\right)=\frac{1}{M}\,\sum_{c\,h=1}^{M}\alpha_{n}^{2}\,\left(c\,h,t\right)$$

where $\alpha_{n}^{2}\,\left(c\,h,t\right)$ is the Fourier coefficient with index *n* of channel *ch* at time *t*. For the right hemisphere $R_{n}^{*}\,\left(t\right)$, the same formula is applied for the left hemisphere electrodes.

We collected resting state data from 16 EEG sites. For the BSI calculation, we discarded the central sites and split the remaining sites in two sets: right and left. For the left hemisphere, the electrodes were: FC3, C5, C3, C1, CP3, and CP1. For the right hemisphere, the electrodes were: FC4, C2, C4, C6, CP2, and CP4.

We processed 8 min of resting state EEG for each participant (in the healthy and stroke groups). We bandpass filtered (1–25 Hz) the whole EEG, and then we cut it in frames of 4 s, with a 2-s overlap. We used a Hamming window to prevent spectral distortion. We used an artifact detection method based on the overflow of the EEG standard deviation on each 4 s window frame. Any frame with more than 1.5 times of the total standard deviation for each channel was rejected from the BSI calculation. We did consider an algorithm for rejection of EOG related artifact, but determined that it would be unnecessary based on analysis of pilot data. The most frontal EEG electrodes are in the row of FCZ; hence, the influence of EOG was small. Furthermore, participants were asked to focus on the screen during the reference period, thus limiting eye movement. Movement related artifacts were found and rejected with our approach based on the standard deviation. Finally, the Fourier coefficient was calculated from the power density estimation using the Welch method.

#### Laterality Coefficient

The raw EEG data recorded during the MI sessions was used to calculate the LC parameter. The LC coefficient was calculated for each session twice: first for trials of MI of the paretic (p) hand and again for trials of the healthy (h) hand. We employed the following formula, where C and I refer to the contralateral and ipsilateral values of the ERD/ERS patterns during the MI.


LC=p/h(C-I)/(C+I)

We followed six steps to calculate C and I:

(1) Band filtering (8–13 Hz or 13–30 Hz) of the EEG signal;
(2) Frame artifact rejection if a sample overflows a threshold based on the median variance among the samples of all the frames;
(3) Laplacian derivation using the surrounding electrodes;
(4) ERD/ERS patterns calculation according to (Graimann et al., 2002);
(5) Summation of all ERD/ERS values from second 2 until the end of the ERD map (second 8); and
(6) Apply the formula to obtain the LC coefficients.

### Assessment Tests

We used 10 tests to assess each patient’s functional capabilities during each of the four assessment sessions. All of these tests are well-established in the scientific literature and clinical practice. These ten tests each measure different aspects of motor function, other motor impairment (tremor and spasticity), cognitive function, sensory discrimination, and self-reported impact. The Supplementary Material provides a complete description of each test.

The scales used for the motor assessment were: Fugl-Meyer Assessment (FMA) for the upper extremity (FMAue) and for the lower extremity (FMAle), Box and Block Test (BBT) and 9 Hole Peg Test (HPT) for the healthy and paretic hand. Each patient completed the BBT and 9HPT tests with both hands to provide an individualized baselne.

The scale used to assess the tremor was the Fahn Tremor Rating Scale (FTRS). We assessed tactile discrimination with the Two Point Discrimination Test (TPDT). The spasticity of the wrists and fingers was assessed with the Modified Ashworth Scale (MAS). The Montreal Cognitive Assessment (MOCA) was used to assess the cognitive status of the patients. The Barthel Index (BI) was used to assess performance in daily life activities.

Patients also completed a self-rated questionnaire (SRQ) to assess pain, function, memory, thinking, mobility and the home and community, and stroke recovery.

### Statistical Analysis

The sample size selection was based on the previous literature (Kaiser et al., 2012). The statistical analyses were performed using MATLAB R2017a (MathWorks Inc., United States). Normality of data was tested using the Shapiro–Wilk test. The statistical test was chosen according to the normality of the sample, the homogeneous of variance (Levene’s or Brown–Forsythe test of equal variance) and sample size. Leven’s test of equal variance was used if normality could be assumed; otherwise, the Brown–Forsythe test of equal variance was used. Descriptive statistics are reported as mean and the standard deviation (SD), or median and the inter-quartile range (IQR) of 0.25 and 0.75.

The correlation tests were chosen according to the results obtained by the Shapiro–Wilk test. When the normality assumption was not rejected, the correlation was made using Pearson’s linear correlation test; otherwise, the correlation was done using Spearman’s rank correlation test.

For the two group comparisons (for example, BSI comparison based on gender), the test was selected based on the normality and homogeneity of variance of the samples and the independence assumption. The unpaired *t*-test was used for comparisons of two independent likely normally distributed groups. For the comparisons before-and after the treatment [Pre2 vs. Post1] with measures from the same population, the paired *t*-test or the Wilcoxon test was used, depending on the normality assumption.

For comparisons between multiple groups, Welch’s ANOVA was used. We used Welch’s ANOVA for comparisons between multiple groups. When Welch’s ANOVA yielded significant results, we conducted a *post hoc* analysis using the Single-step Games-Howell test.

A correction for multiplicity (i.e., multiple hypotheses testing) was not utilized, because no final conclusion and decision on the correlation of qEEG parameters and functional scales is drawn. Similarly, while a statistical analysis concerning the functional scales before and after BCI therapy was carried out, the goal of this paper is not to establish definitive proof on the efficacy of BCI-based therapy. Concordantly, we did not correct for multiplicity in this statistical analysis either (Bender and Lange, 2001). Additionally, Hurlbert and Lombardi (2012) recommend carrying out statistical tests without any adjustments for multiple hypothesis. This recommendation stems from the reasoning that the true probability of type I errors occurring is very small, because most null hypotheses can be expected to be false based on prior knowledge (Hurlbert and Lombardi, 2012). In the present case, one can reasonably accept this premise, based on the available literature discussed in the section “Introduction.”

## Results

### Participants’ Baselines

Thirty-two healthy subjects were enrolled in the study, with 13 males and 19 females. The mean age in the healthy group was 42.3 years (*SD* = 15.4). Thirty-four stroke patients participated (excluding two who dropped out). Twenty-two of the patients were male (64.7%), and the other 12 stroke participants were female (35.3%). The stroke patients’ mean age was 65.3 years (*SD* = 14.4); this difference in age will be addressed at a later stage of this analysis.

Table 1 shows the participants’ baselines. The stroke participants were classified in three groups based on their stroke diagnosis: Cortical, Subcortical, Cortical + Subcortical. The most common type of stroke was Subcortical with 17 patients (50.0%), followed by Cortical+Subcortical with 12 patients (35.3%) and Cortical with 5 patients (14.7%). Twenty-seven of these patients were in the chronic phase (79.4%), and only 7 in the subacute phase (20.6%). Twenty three patients had a stroke in the right hemisphere (67.7%), and the stroke was in the left hemisphere in 11 patients (32.4%).

**TABLE 1:** Participants’ baselines.

Group	*n*	Age (y)	*SD*	Male	Female
Healthy	32	42.3	15.4	13	19
Patient	34	65.3	14.4	22	12
Cortical	5	57.6	27.3	4	1
Subcortical	17	66.4	12.7	9	8
Cortical + Subcortical	12	67.0	09.4	9	3

### Brain Symmetry Index (BSI)

#### BSI Differences Between Age Groups

**FIGURE 3:**
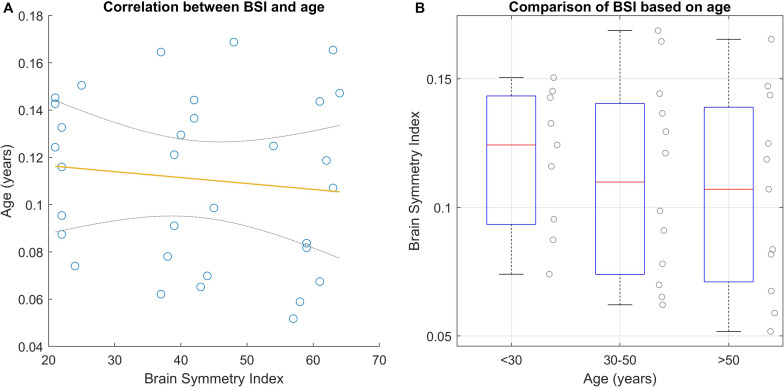
BSI analyses based on age. **(A)** Shows the correlation between age and BSI using Pearson’s method. The yellow line is the regression line and the gray lines are the CI at 95%. **(B)** Shows the BSI results based on three clusters.

To date, there is no evidence to demonstrate the variability of BSI with age. We performed a statistical analysis using the rEEG data from the healthy subjects. The data follows a normal distribution according to the Shapiro–Wilk test (*P* = 0.117). We explored the relationship between BSI and age using Pearson’s method and One-way ANOVA. Figure 3A shows that the Pearson’s correlation did not show significant correlation between BSI and age (ρ = −0.110, *P* = 0.548). Subsequently, we compared BSI across age groups (under 30 years, between 30 and 50 years and over 50 years). The variance of each group, using Levene’s test, did not show significant results (df = 29.00, *F* = 1.338, *P* = 0.278). The analysis of variance shows that there is no significant difference in the BSI parameter based on the three age groups (*F* = 0.3843, *P* = 0.684). See Figure 3B and Table 2.

**TABLE 2:** Results of BSI-based age analysis.

One-way ANOVA					
	SS	Df	MS	*F*	*P*
Groups	0.000983	2	0.000492	0.3843	0.6844
Error	0.0371	29	0.0013		
Total	0.0381	31			

#### BSI Based on Gender

**FIGURE 4:**
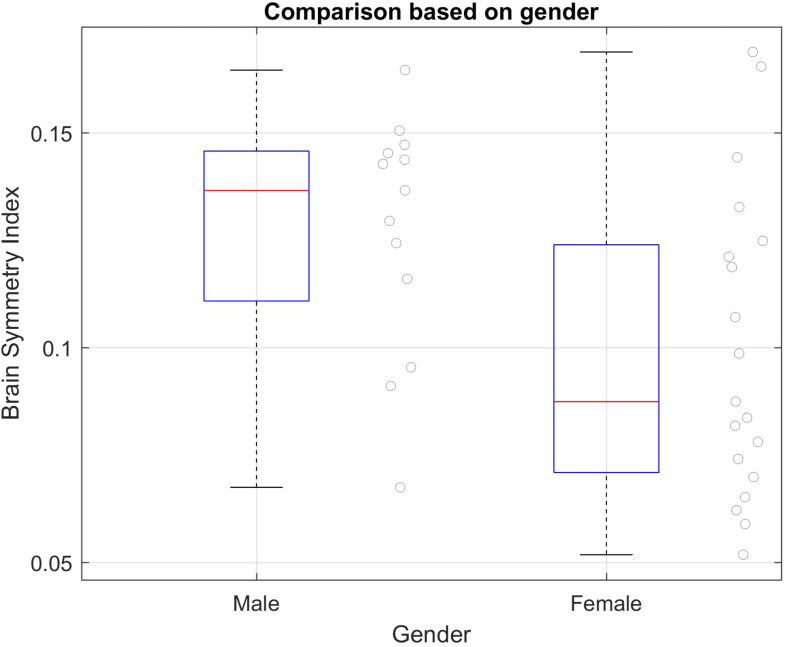
BSI subgroup analyses based on gender in the healthy group. Mean and SD of each group: Male = 0.1272 (*SD* = 0.0278); Female = 0.0997 (*SD* = 0.0357). Significant difference between groups using unpaired *t*-test; *t*-value = | 2.333| *P* = 0.027.

Figure 4 presents the results of this subgroup analysis. Both groups have similar variance (Levene’s test results: df = 30.00, *F* = 1.733, *P* = 0.198). The result of this analysis shows that there is a statistical difference in BSI based on gender, according to the unpaired *t*-test (*t*-value = | 2.333|, *P* = 0.027).

#### BSI Between Groups

**FIGURE 5:**
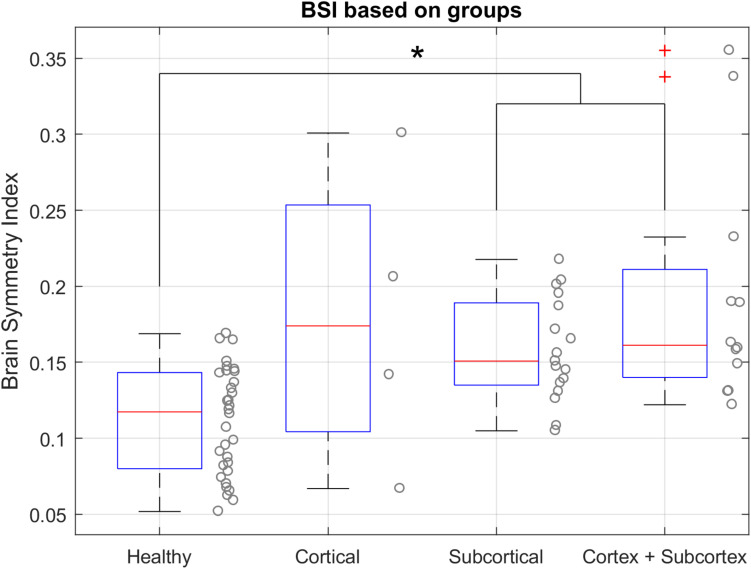
BSI values in resting state with open eyes for each group. (*) Indicates the significant differences based on the Games-Howell test. The BSI of the healthy group is significantly different from the subcortical group and cortex + subcortex group. Healthy group median = 0.1173, IQR = 0.0799 – 0.1432. Cortical group = 0.1739, IQR = 0.1043–0.2534. Subcortical group = 0.1507, IQR = 0.1349–0.1890. Cortex + Subcortex group = 0.1612, IQR = 0.1400–0.2110.

Since the results obtained in the BSI based on age did not show significant differences in the healthy group, we compared the BSI between groups (stroke and healthy) despite the age difference. We first analyzed the resting state data collected during the assessments, consisting of 136 assessment sessions from 34 stroke patients and 32 EEG recordings from 32 healthy subjects. We calculated the BSI of each assessment (Pre1, Pre2, Post1, Post2) from each patient’s rEEG data. For this analysis, we used the median of Pre2 and Post1. In one case, the BSI could not be calculated due the corruption of the EEG data recordings (this patient belongs to Cortical group). The first step was the normality testing of each group. The first three datasets did not attain significance using the Shapiro–Wilk Test, hence, a normal distribution can be assumed. Group 4 (cortex + subcortex) is the only one that is not normally distributed. The equality of the variances cannot be assumed (Levene’s Test results: df = 61.00, *F* = 5.798, *P* = 0.001). We used Welch’s ANOVA test to compare the BSI parameter across the four groups, because this method is reasonably robust to deviations of normality, when the variances are substantially different and even if the sample sizes are unequal (Kohr and Games, 1974; Tomarken and Serlin, 1986). Table 3 summarizes results from each group. Welch’s ANOVA test found an associated probability of *P* = 0.003 and *F* = 8.929, so the hypothesis of equal sample means was rejected. As the Welch’s ANOVA test showed significant results, the Games-Howell test was used to complete the analysis. The single-step Games-Howell test (see Table 4) shows significant differences between group 1 (healthy group) and both group 3 (subcortex group) and group 4 (cortex + subcortex group). Figure 5 shows the BSI values for each group.

**TABLE 3:** BSI analysis summary statistic.

Group	Size	Mean	Variance
1	32	0.1109	0.0012
2	4	0.1789	0.0098
3	17	0.1580	0.0011
4	12	0.1931	0.0061

*Group numbers: 1 – Healthy, 2 – Cortical, 3 – Subcortical, 4 – Cortical + Subcortical.*

**TABLE 4:** Single-step Games-Howell test.

Comparison		Delta	*SE*	Df	*P*	*H*	lb	ub
1	2	−0.068	0.045	4.157	0.505	0	−0.246	0.110
1	3	−0.047	0.010	33.870	0.001	1	−0.075	−0.019
1	4	−0.082	0.023	12.708	0.019	1	−0.151	−0.013
2	3	0.021	0.045	4.275	0.965	0	−0.156	0.198
2	4	−0.014	0.050	6.171	0.992	0	−0.185	0.156
3	4	−0.035	0.024	13.937	0.481	0	−0.105	0.035

*Result of group comparison based on BSI values using Games-Howell test. The first column shows the group code; 1 – Healthy group, 2 – Cortical group, 3 – Subcortical group, 4 – Cortex + Subcortex group. The column ‘H’ shows the significant (*H* = 1) and non-significant (*H* = 0) differences at alpha level, and the column ‘*P*’ shows the significance level of these comparisons.*

#### Correlations Between BSI and Functional Tests

**FIGURE 6:**
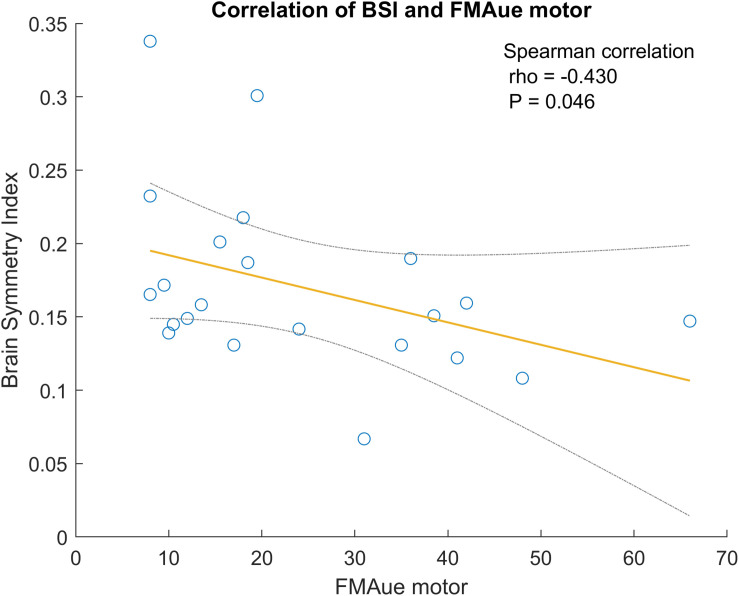
Correlation between BSI and FMA upper extremity. The gray lines are the confidence interval at 95%.

Figure 6 shows a significant correlation between BSI and patients’ outcomes on the FMAue scale. The correlation coefficient of this relationship is −0.430 and *P* = 0.046. Lower BSI values are related to better functionality.

### Laterality Coefficient

The LC was calculated separately for the MI tasks of the healthy (LCh) and paretic (LCp) hand. We calculated the LC for the alpha (LChα and LCpα) and beta (LChβ and LCpβ) bands. We explored the LC (α and β) between groups (Cortical, Subcortical, and Cortical + Subcortical), and found no significant differences of LC between groups (Welch ANOVA, *F* = 0.36, *P* = 0.7033).

In this part of the analysis, we correlated the LC mean of the 25 BCI therapy sessions against the mean of the results from motor tests collected in the Pre2 and Post1 assessment visits. The Shapiro–Wilk Test shows that the data are likely not normally distributed at alpha level. The Spearman test has been used for the correlation analysis. Table 5 shows the correlation’s results of LC against the functional scales.

**TABLE 5:** Significant correlations between LC and functional scales using Spearman Correlation are colored red.

Scale		Laterality Coefficient							
		α				β			
		LC_*h*_		LC_*p*_		LC_*h*_		LC_*p*_	
Name	Side	ρ	*P*	ρ	*P*	ρ	*P*	ρ	*P*
BI	−	−0.260	0.138	0.058	0.743	−0.154	0.383	0.184	0.296
FTRS	Healthy	−0.038	0.829	0.105	0.555	−0.093	0.600	0.060	0.734
	Paretic	0.450	0.008	−0.245	0.162	0.336	0.052	−0.490	0.003
MAS	Wrist	0.076	0.670	−0.216	0.220	−0.116	0.514	0.034	0.848
	Fingers	0.237	0.176	−0.262	0.134	0.109	0.539	−0.099	0.579
BBT	Healthy	0.102	0.566	−0.154	0.386	0.059	0.741	−0.141	0.425
	Paretic	−0.616	<0.001	0.354	0.043	−0.418	0.016	0.569	0.001
9HPT	Healthy	−0.042	0.813	0.036	0.839	−0.167	0.345	0.186	0.291
	Paretic	0.536	0.236	−0.357	0.444	0.714	0.088	−0.607	0.167
TPDT	Thumb *H*	−0.169	0.340	0.031	0.862	0.038	0.830	−0.005	0.979
	Index *H*	−0.010	0.956	0.041	0.820	−0.053	0.765	0.157	0.374
	Thumb *P*	0.000	0.999	−0.067	0.746	−0.139	0.499	0.152	0.459
	Index *P*	0.065	0.751	−0.079	0.701	−0.082	0.689	−0.125	0.543
FMAue	–	−0.706	<0.001	0.400	0.019	−0.440	0.009	0.384	0.025
FMAle	–	−0.601	0.006	0.271	0.261	−0.252	0.298	−0.057	0.817
SRQ	Pain	0.287	0.100	−0.157	0.374	0.095	0.591	−0.115	0.518
	Function	−0.427	0.012	0.316	0.069	−0.488	0.003	0.447	0.008
	Memory	−0.068	0.704	−0.130	0.465	−0.226	0.198	−0.033	0.855
	Mobility	−0.216	0.219	−0.034	0.849	−0.150	0.396	0.033	0.855
	Recovery	0.065	0.717	−0.205	0.245	0.083	0.642	0.061	0.732
MOCA	–	0.005	0.982	−0.032	0.884	−0.232	0.288	0.043	0.847

#### Alpha Band

**FIGURE 7:**
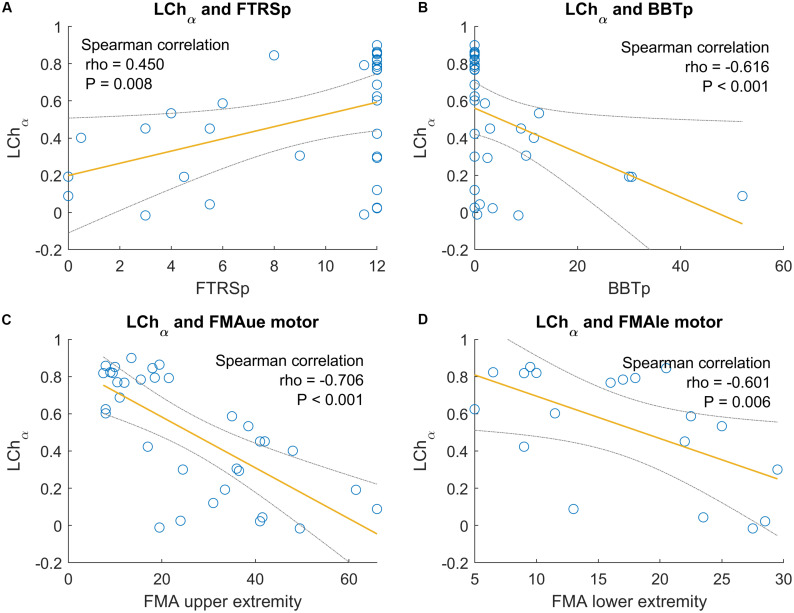
Significant correlations between LC of the paretic hand in alpha band and functional scales. The gray lines are the confidence interval at 95%. **(A)** Correlation between LChα and BBT of the paretic hand. **(B)** Correlation between LChα and FTRS of the paretic hand. **(C)** Correlation between LChα and FMAue of the motor part. **(D)** Correlation between LChα and FMAle of the motor part.

The LC calculated during the MI task with the healthy hand (LChα) is the parameter that shows the highest correlation with functional scales. In general terms, the results show that LC values near 0 are related to better functionality and less tremor in the paretic upper extremity (see Figure 7).

Tremor of the paretic hand assessed by FTRS shows a significant correlation with the LChα. The correlation coefficient is positive (ρ = 0.450 and *P* = 0.008). Thus, low degrees of tremor are related to LChα values near to 0.

In the BBT of the paretic hand, there is a stable correlation with all the LC parameters and bands. Here, the LChα shows a correlation but with a negative sign. The correlation is strong (ρ = −0.616 and *P* < 0.001). This correlation shows that good scores in the grasp ability, as assessed by BBT, are related to low values of LChα.

Also, the LChα parameter showed significant correlations with the FMA upper and lower extremity. The FMAue correlation has a stronger correlation coefficient (ρ = −0.706 and *P* < 0.001) than the FMAle (ρ = −0.601 and *P* = 0.006). The correlation coefficient is negative in both cases, and these results are consistent with the other relationships explained above – better motor function in the lower and upper extremity, as assessed by FMA, is related to LChα values near to 0.

Finally, the LChα is also correlated with the function score of the SRQ (ρ = −0.427 and *P* = 0.0212). The function score of SRQ is based on the subjective opinion of the patient doing different motor tasks. The negative correlation shows that good scores in the function score of SRQ are related to low values of LChα.

Other similar correlations with opposite signs have been found for the LCpα. In this case, LCp values near to 0 are related to better performance in the FMAue score (ρ = 0.400 and *P* = 0.019) and also in the BBT of the paretic hand (ρ = 0.354 and *P* = 0.043).

#### Beta Band

LChβ and LCpβ also presented some interesting correlations with the functional scales. In general, the correlations found in this frequency band are weaker than the correlations found in the alpha band. The low tremor degree in the paretic hand assessed by FTRS (higher scores in this scale) is correlated with values near to 0 in LCpβ (ρ = −0.490 and *P* = 0.003). The good grasp ability in the paretic hand, assessed by BBT (BBT_p), is also correlated with low values of LChβ (ρ = −0.418 and *P* = 0.016) and values near 0 in LCpβ (ρ = 0.569 and *P* = 0.001). The general motor function of the upper extremity, assessed by FMA, is also correlated with LChβ (ρ = −0.440 and *P* = 0.009), and with LCpβ (ρ = 0.384 and *P* = 0.025).

Finally, the last significant correlation is between the function scale part of SRQ and LChβ (ρ = −0.488 and *P* = 0.003) and LCpβ (ρ = 0.447 and *P* = 0.008). Again, the best functionality is related to values near to 0 of both LC parameters. All correlation results regarding the LC and functional scales can be seen in Table 5.

### Assessment Tests Before and After BCI Treatment

The results in this section summarize differences from the Pre2 to Post1 assessments across different tests. We used the Wilcoxon signed rank test for statistical analysis, since the data did not present a normal distribution (see Table 6). The improvement of each scale is presented using the median and IQR, and the mean and SD are also provided if differences are significant.

**TABLE 6:** Changes in the functional scales.

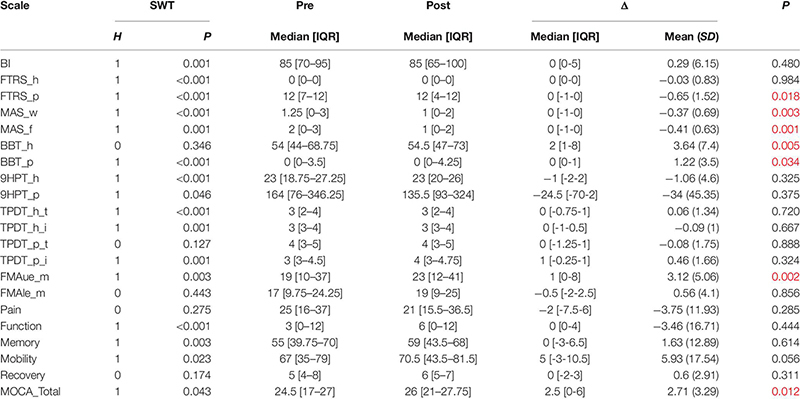

*The first column shows the results of the Shapiro–Wilk Test (SWT), to assess the normality of the dependent variable. The last column (*P*) presents the probability results of the paired test, with statistically significant differences colored red.*

#### Fugl-Meyer Assessment

The FMAue test has a score range of 0–66. One of the 34 patients had only slight hemiparesis and attained the maximum FMAue score in the pre-assessment. The Wilcoxon signed rank test shows that there is a significant improvement in FMAue after the therapy (ΔFMAue = 1 [0–8], *P* = 0.002). The mean improvement is 3.12 (*SD* = 5.1). Twenty one patients (61.8%) improved at least 1 point in the FMA score. Among patients who improved, the mean improvement was 5.76 points (*SD* = 4.61). Six patients (17.7%) decreased at least 1 point in FMA score, and the mean decrease in this group was −2.5 points (*SD* = 1.76). The remaining 6 patients (17.7%) had an improvement equal to 0.

#### Barthel Index

The BI did not show significant improvements after the therapy (ΔBI = 0 [0–5], *P* = 0.480). The BI score decreased in 7 patients (20.6%), 11 patients (32.4%) reported positive changes in the BI after the therapy, and 16 patients (47.1%) did not show changes in this parameter.

#### Fahn Tremor Rating Scale

The FTRS for the healthy hand (FTRS_h) did not show a significant difference before versus after the BCI therapy (ΔFTRS_h = 0 [0–0], *P* = 0.984). The FTRS in the paretic hand (FTRS_p) did show a significant improvement (ΔFTRS_p = 0 [−1-0], *P* = 0.018). The mean improvement of FTRS_p is −0.65 (*SD* = 1.5). Thirty two of the 34 patients (94.1%) reported some degree of tremor in the paretic hand (FTRS_p) before the therapy. After the therapy, 10 of these 32 patients (31.3%) exhibited a decreased tremor in the paretic hand. One of these 32 patients (3.1%) showed an increase of tremor after the therapy. The other patients did not report any changes.

#### Modified Ashworth Scale

The MAS scale showed a statistical reduction of the spasticity in the wrist [ΔMAS_w = 0 [-1-0], *P* = 0.003, mean improvement −0.37 (*SD* = 0.69)], and in the fingers [ΔMAS_f = 0 [-1-0], *P* = 0.001, mean improvement −0.41 (*SD* = 0.63)]. Twenty three of the 34 patients (67.7%) reported some spasticity in the wrist (MAS > 0), and 25 patients (73.5%) reported some spasticity in the fingers. Twelve of the 23 patients (52.2%) who reported wrist spasticity prior to therapy reported a decrease after therapy. Fourteen of the 25 patients (56.0%) who reported finger spasticity prior to therapy reported a decrease after therapy.

#### Box and Block Test

We observed a statistical improvement of BBT in the healthy hand [ΔBBT_h = 2 [1–8] and *P* = 0.005, mean improvement 3.64 (*SD* = 7.4)]. The changes in the paretic hand are also significant [ΔBBT_p = 0 [0–1] and *P* = 0.034, mean improvement 1.22 (*SD* = 3.5)]. Ten patients (29.4%) improved the BBT score with the paretic hand, 2 patients (5.89%) decreased the BBT score with the paretic hand, and 22 patients (64.7%) did not change from the initial BBT score. In three cases (8.8%), it was impossible to perform the BBT before the therapy due to the severity of the motor impairment, but after the therapy, these patients could move at least 1 block in the BBT.

#### 9HPT

The 9HPT in the paretic hand is one of the most commonly used tests of grasp function. Only five patients (14.7%) could perform the test before the therapy, and six patients (20.6%) could perform this test after the treatment. No significant improvements were observed after the therapy in the healthy hand (Δ9HPT_h = −1 [-2-2], *P* = 0.325), or in the paretic hand (Δ9HPT_p = −24.5 [-70-2], *P* = 0.375). The results show that the time in the healthy hand has slightly decreased, and in the affected hand the decrease in time was great.

#### Two Point Discrimination Test

This test did not show significant changes before vs. after the therapy in the thumb or index of the healthy hand (ΔTPDT_h_t = 0 [-0.75-1], *P* = 0.720; ΔTPDT_h_i = 0 [-1-0.5], *P* = 0.667). The paretic hand did not show a significant improvement either (ΔTPDT_p_t = 0 [-1.25-1], *P* = 0.888; ΔTPDT_p_i = 1 [-0.25-1], *P* = 0.324). Eight patients (23.5%) improved the discrimination between two points in the healthy thumb by at least 1 mm, and nine patients (26.5%) improved in the healthy index finger. Six patients (17.7%) improved in the TPDT at least by 1 mm in the paretic thumb, and three patients (8.8%) reported at least 1 mm of improvement in the paretic index.

#### SRQ

Sixteen patients (47.1%) reported at least 1 point of pain reduction, and 11 patients (32.4%) reported at least 1 point of pain increase. Thirteen patients (38.2%) reported an improvement in the ability to perform ADLs, and six patients (17.7%) reported a decrease in ADL performance. Eleven patients (32.4%) reported an improvement in the memory part, and 11 patients (32.4%) reported a decrease in memory. Sixteen patients (47.1%) reported an improvement in the mobility part of the questionnaire, while 11 patients (32.4%) reported a decrease in mobility. Finally, 12 patients (35.3%) reported a better general recovery after BCI therapy, and 10 patients (29.4%) reported a worse recovery after BCI therapy. There are no significant changes in any part of SRQ (ΔPain = −2 [-7.5-6], *P* = 0.285; ΔFunction = 0 [0-4], *P* = 0.444; ΔMemory = 0 [-3-6.5], *P* = 0.614; ΔMobility = 5 [-3-10.5], *P* = 0.056; ΔRecovery = 0 [-2-3], *P* = 0.311).

#### MOCA

The comparison between before and after the therapy showed a significant improvement in cognitive function, ΔMOCA = 2.5 [0-6], *P* = 0.012, with a mean improvement of 2.71 (*SD* = 3.29). Ten patients (29.4%) improved by at least one point after therapy, and the MOCA score decreased in two patients (5.9%). The remaining patients reported no change.

## Discussion

The objective of this experiment was to explore how two EEG-based parameters relate to different facets of stroke diagnosis and functional prognosis during BCI-based stroke rehabilitation therapy. The BSI was derived from EEG data recorded during the assessment visits in the resting state, while the LC was based on EEG data recorded during MI exercises.

### BSI, Age, and Gender

The analysis of the BSI in healthy subjects based on age suggests that this parameter does not change with age. However, this issue needs to be further explored in larger studies. The results show significant difference in BSI based on gender; males usually have higher BSI values than females. These results could help our understanding of the BSI parameter in healthy conditions, improve detection of pathological values correlated with different brain affectations that can help with diagnosis of stroke and other conditions, and support further research involving gender differences.

### BSI and Stroke Diagnoses

Stroke patients were divided into three different groups based on stroke location; Cortical, Subcortical and Cortical + Subcortical. The Cortical group was the smallest group with only five patients and exhibited the highest BSI variability. Prior work found similar results, with an almost identical boxplot distribution but a smaller sample size (Agius Anastasi et al., 2017). Our results show that healthy participants had significantly lower BSI values than stroke patients of the Subcortical group (*P* = 0.001) and the Cortex + Subcortex group (*P* = 0.019); see Table 4 and Figure 5. The high variability in the Cortical group may be due to small size of this subgroup. Moreover, in these patients the location of the lesion is very peripheral, and most of the neural activity observable via EEG originates from the cortex; consequently, the aberrant neural activity is more apparent in these patients than in the ones with other stroke locations.

Hence, despite the high variability in the Cortical group, the BSI parameter did differ significantly between the healthy control vs. stroke groups Subcortical and Cortex + Subcortex. Results were consistent with prior work (Agius Anastasi et al., 2017). With further research, the BSI could become a tool to support stroke diagnosis, including stroke location and severity.

### BSI and Functional Impairment

We also analyzed the correlations between the values of BSI and the patient’s functional state (Figure 6). The most noteworthy correlations observed showed that patients with lowest BSI have better motor function in the upper extremities (FMAue). The correlation between BSI and FMAue was also observed in prior studies; lower BSI values were correlated with higher functionality in the upper extremity (Agius Anastasi et al., 2017). Thus, the BSI could be a useful parameter to assess functional impairment during stroke assessment and rehabilitation.

### LC in Alpha Band

We calculated the LC using the event-related synchronization and desynchronization patterns generated during the MI task (Kaiser et al., 2012). The LC is derived in a similar manner as the BSI, but the LC yields results from −1 to 1. We calculated the LC in two frequency bands, 8–13 Hz (α band, mu frequency rhythm) and 13–30 Hz (β band) and found the most relevant results in the alpha band. In general, LC values calculated during the MI tasks with the healthy hand (LCh) were between 0 and 1, while LC values of the paretic hand MI tasks (LCp) were between −1 and 0. The LCh in alpha band presented numerous significant correlations with functional scales. We also observed most of these significant correlations with the LCp parameter, but with the opposite sign.

The LC values for the healthy hand presented noteworthy correlations with four dependent variables. LCh values near 0 were related with a higher BBT score in the paretic hand, which indicates better grasp function (ρ = −0.616 and *P* < 0.001). The LCh was also significantly correlated with tremor, assessed by FTRS. Participants with LCh values near 1 tended to have a higher FTRS score (reflecting greater tremor) in the paretic hand (ρ = 0.450 and *P* = 0.008). Finally, the LCh parameter was significantly correlated with the FMAue and FMAle. LCh values closer to 0 reflect better motor functionality for the upper extremity (FMAue, ρ = −0.706 and *P* < 0.001) and for the lower extremity (FMAle, ρ = −0.601 and *P* = 0.006).

The correlations between the LCp and the functional scales are less common than the LCh. This could occur because the affected hemisphere does not present a normal activation pattern due the stroke, but the healthy hemisphere maintains the normal patterns of desynchronization during the ipsilateral motor movements (originated in the affected hemisphere). The ERD/ERS patterns observed in the healthy hemisphere should be more stable than the ERD/ERS patterns observed in the affected side of the brain.

### LC in Beta Band

The LC calculated in the β band showed similar correlations (see Table 5). Interestingly, LCβ shows significant correlations with the scales where more mental concentration is required (FTRS and BBT). In both scales, values near 0 in LCβ (healthy and paretic) are correlated with better grasp ability and less tremor. Other studies showed correlations between the EEG activity in beta band and concentration (Janssen et al., 2017; Kiiski et al., 2020).

### Clinical Improvements Before vs. After BCI Therapy

The main objective of the study was not to demonstrate the efficacy of the BCI system in neurorehabilitation, nor to compare BCI-based therapy to other forms of therapy. The relationship between BCI stroke therapy and functional outcomes has been addressed in numerous studies (Ramos-Murguialday et al., 2013; Pichiorri et al., 2015; Remsik et al., 2016; Biasiucci et al., 2018; Cho et al., 2018). However, we would like to add to the existing literature by discussing our observations.

FMAue was the primary measure of motor function in this study. When assessing the motor function of the upper extremity by FMAue, we found the most important significant clinical improvement (ΔFMAue = 1 [0-8] and *P* = 0.002). On average, the stroke patients improved by 3.21 points (*SD* = 5.1) in the FMAue with the BCI therapy. After the therapy, the patients also presented a significant reduction in tremor (FTRS), spasticity (MAS), and increase on the grasp ability (BBT) and in the cognitive state (MOCA).

In general, the first sign that patients reported during the therapy was a reduction in spasticity, followed with improvement in motor function. The reduced spasticity may drive the improved range of motion and reduced tremor, something that can explain the improvements on FTRS, BBT and FMAue.

The MOCA scale also showed significant improvement, which may be related to the need for concentration during the BCI sessions in order to get positive feedback. The patients have to learn to maintain concentration during the sessions to improve their motor skills using BCI.

## General Discussion

The BSI parameter can be calculated in real-time using portable and practical EEG tools, and thus could be used during stroke diagnosis or ongoing monitoring of patients’ brain activity during stroke rehabilitation and recovery. More broadly, the BSI and LC parameters might contribute to other neurological assessments and ongoing monitoring of brain damage and recovery.

One limitation of this study is the absence of a healthy group that performed the same BCI training as the patient group, which prevents us from comparing LC between these groups. The study may also be limited by the unequal numbers of participants across the three stroke subgroups, and additional work is needed to identify any age differences between the control and stroke groups. Overcoming these limitations will require substantial additional work with more participants in a larger study, which we are currently exploring. Additionally, a great number of hypotheses tests were carried out in the present study. Therefore, results referred to as “statistically significant” may have been obtained by chance and we strongly recommend considering the obtained *p*-values and effect sizes when interpreting the results of this study.

In addition to broader work with more participants, future research could: explore variants of the different measures that we used that might be more informative; identify correlations with other types of diagnoses and therapies relating to motor (and perhaps other) impairment and recovery; evaluate these and other parameters in tandem with other methods to treat stroke, such as medications or non-invasive brain stimulation; measure long-term changes via longitudinal follow-up assessments; and compare the utility of these measures to other tools.

## Data Availability Statement

The datasets for this manuscript are not publicly available because: Patients’ data need to be treated according to current data protection laws and ethical guidelines. Requests to access the datasets should be directed to MS-R, sebastian@gtec.at.

## Ethics Statement

The studies involving human participants were reviewed and approved by Ethikkommission des Landes Oberösterreich. The patients/participants provided their written informed consent to participate in this study.

## Author Contributions

MS-R participated in the data acquisition, performed the analysis, and did the main contribution to the manuscript writing. EU supervised the data analysis and assisted in the manuscript preparation. RO supervised the whole process, data acquisition, analysis, and manuscript revision. JD-F supervised the signal processing methods. NM participated to the data acquisition from stroke participants. CM-P did the acquisition of healthy subjects. SS participated in the data analysis. BA provided scientific input and contributed to the manuscript writing. CG supervised the whole project and reviewed the manuscript. WC contributed to the data analysis.

## Conflict of Interest

MS-R, RO, JD-F, CM-P, and CG are employed at g.tec medical engineering Spain SL and CG is CEO of g.tec medical engineering Spain SL and g.tec medical engineering GmbH. This study was conducted primarily as a component of a Ph.D. program, and our motivation for conducting the study to explore different methods was entirely scientific. However, the described algorithms may be commercialized by g.tec medical engineering GmbH in the future. The remaining authors declare that the research was conducted in the absence of any commercial or financial relationships that could be construed as a potential conflict of interest.
