# Corrigendum: EEG biomarkers related with the functional state of stroke patients

**DOI:** 10.3389/fnins.2022.1032959

**Published:** 2022-09-23

**Authors:** Marc Sebastián-Romagosa, Esther Udina, Rupert Ortner, Josep Dinarès-Ferran, Woosang Cho, Nensi Murovec, Clara Matencio-Peralba, Sebastian Sieghartsleitner, Brendan Z. Allison, Christoph Guger

**Affiliations:** ^1^Department of Physiology, Universitat Autònoma de Barcelona, Barcelona, Spain; ^2^g.tec Medical Engineering Spain SL, Barcelona, Spain; ^3^Data and Signal Processing Research Group, Department of Engineering, University of Vic - Central University of Catalonia, Vic, Spain; ^4^g.tec Medical Engineering GmbH, Schiedlberg, Austria; ^5^Department of Cognitive Science, University of California at San Diego, La Jolla, CA, United States

**Keywords:** brain-computer interface, motor imagery, EEG, rehabilitation, Brain Symmetry Index, laterality coefficient

In the original article, we neglected to include the following funding information:

“This work was supported by the European Union's Horizon 2020 Research and Innovation Programme under the Marie Skłodowska-Curie grant agreement No. 839234 (DOC-Stim).”

The authors apologize for this error and state that this does not change the scientific conclusions of the article in any way. The original article has been updated.

## Publisher's note

All claims expressed in this article are solely those of the authors and do not necessarily represent those of their affiliated organizations, or those of the publisher, the editors and the reviewers. Any product that may be evaluated in this article, or claim that may be made by its manufacturer, is not guaranteed or endorsed by the publisher.

