# Supplementary material for: EEG Biomarkers Related With the Functional State of Stroke Patients
Source: Front Neurosci. 2020 Jul 7;14:582. doi: 10.3389/fnins.2020.00582 (PMC7358582; doi:10.3389/fnins.2020.00582)
Supplement: Supplementary file 1 [file Data_Sheet_1.PDF]

Supplementary material I - Detailed description of the functional scales used in the study:

Fugl-Meyer Assessment (FMA): The primary measure of this study is the Fugl-Meyer Assessment for the upper extremity (FMAue). This scale contains values between 0 to 66 points, where a score of 0 reflects no motor function in the upper extremities and 66 points indicates normal function.

We also used the the Fugl-Meyer Assessment for the lower extremity (FMAle) to assess the motor function of the lower limb. The FMAle scale ranges from 0 points (no lower-limb functionality) to 34 points (normal function).

Box and Block Test (BBT): In the BBT, the patient must move 100 small blocks from one container to another, while avoiding an obstacle between them. The outcome is measured in the number of blocks moved within a specific time (one minute).

9 Hole-Peg Test (9HPT): Like the BBT, this test measures how patients can grasp and move small objects from one area to another. In the 9HPT, each patient must pick up nine small pegs from a box and place them in 9 holes and then remove them, while only holding at most one peg at any time. The outcome is measured in completion time; a shorter completion time indicates higher motor function. Like the BBT, some patients with more severe disabilities may be unable to perform this task at all.

Fahn Tremor Rating Scale (FTRS): This test is designed to measure each patient's tremor. The patient performs the test with both hands, resulting in a score between 0 points (no tremor) to 12 points (maximum tremor) for each hand.

Modified Ashworth Scale (MAS): The MAS is used to assess each patient's spasticity. The minimum score is 0 points (no spasticity) and maximum score is 4 points (passive range of motion is totally restricted for the spasticity).

Montreal Cognitive Assessment (MOCA): The MOCA was designed as a rapid screening instrument for mild cognitive dysfunction. It assesses different cognitive domains: attention and concentration, executive functions, memory, language, visual construction skills, conceptual thinking, calculations, and orientation. The maximum possible score is 30 points, and a score of 26 or above is considered normal.

Two Point Discrimination Test (TPDT): The TPDT was employed to assess finger sensitivity. This test evaluates the minimum distance between two points that patients can discriminate. The clinician touches the patient's fingertips with two very sharp tips (like needles) and asks if the patient can feel both points. If so, the clinician reduces the distance between the two tips and repeats the test until the patient reports feeling only one point. Patients who can feel two distinct tips that are closer together have greater finger sensitivity.

Self-Rated Questionnaire (SRQ): The SRQ is a questionnaire with five parts: Pain (0-70 points); Function (0-70 points); Memory and thinking (0-70 points); Ability to be mobile at home and in the community (0-90 points); Stroke recovery (0-10 points). Each of these parts has descriptions of different tasks. The patient estimates the difficulty in performing the task on a scale from 0-10,

where 0 means ‘unable to do’, and 10 means ‘no difficulty’. The scale is different for the Pain part, where 0 means ‘none’ and 10 means ‘extreme’.

Barthel Index (BI): This scale is widely used to evaluate how well patients can perform activities of daily living (ADL). Like the SRQ, it is a questionnaire that measures the impact of stroke from the patient’s personal, subjective perspective. The minimum score is 0 points, which indicates that the patient cannot perform any ADLs. The maximum score is 100, in which the patient can perform all ADLs well.
